# An Integrative Review on Teen Distracted Driving for Model Program Development

**DOI:** 10.3389/fpubh.2019.00111

**Published:** 2019-05-03

**Authors:** Sherrilene Classen, Sandra M. Winter, Charles Brown, Jane Morgan-Daniel, Shabnam Medhizadah, Nithin Agarwal

**Affiliations:** ^1^Department of Occupational Therapy, University of Florida, Gainesville, FL, United States; ^2^University of Florida Transportation Institute, Gainesville, FL, United States; ^3^Health Science Center Libraries, University of Florida, Gainesville, FL, United States

**Keywords:** distracted driving, adolescent, United State of America, safety management, accidents and fatalities, attention maintenance, hazard mitigation, hazard anticipation

## Abstract

Distracted driving, especially driver inattention, is associated with high levels of crash-related fatalities and injury. Teen novice drivers are one of the groups most likely to drive distracted and to suffer its consequences. Teens have a higher risk of engaging in texting or secondary tasks, e.g., eating while driving. Distracted driving interventions to date aim to improve teen and societal safety, but few have achieved effectiveness. A need exists for effective evidence-based distracted driving interventions. We used an integrative review to identify rigorous evidence, and inform the development of a teen distracted driving educational intervention. This five-step review included: identifying the research problem; collecting literature; evaluating literature; synthesizing data; and presenting results. We searched 6 databases, identifying 185 articles. Following three rounds of inclusion screening (title, abstract, and full-text), captured according to a PRISMA flow chart, 17 studies met inclusion. We categorized these studies, conducted in the U.S., as five intervention types that used approaches including presentations, videos or instructional programs, education or training programs, driving simulator training, in-vehicle monitoring or feedback, and integrated programs. Study designs included randomized controlled trials pre-post, quasi-experimental, and experimental designs with prospective longitudinal cohorts. The studies were heterogeneous in design, intervention and outcome. However, three core themes emerged across studies: i.e., hazard awareness, hazard mitigation and attention maintenance are primary critically necessary skills to prevent distracted driving; engaging a parent or adult as a partner in the intervention process from classroom to car contributed to the effectiveness of the intervention; and leveraging technology in training enhanced the effectiveness of the intervention. Study limitations pertained to a focus on short-term effects; sampling distributions that did not account for gender, age, race, and/or ethnicity; types of interventions; and bias. The limitations affect the generalizability of included study findings and, potentially, the review findings, as they may not apply to populations or contexts outside those synopsized. Strengths included our team's expertise in conducting evidence-based reviews, support of a health science librarian, and use of international review guidelines. As an outcome, we are applying findings of the integrated review to develop a computer-based training addressing teen distracted driving.

## Introduction

Distracted driving, a specific category of driver inattention, is a public health epidemic associated with high levels of crash-related fatalities and injury (1, 2). Distracted driving has high personal, societal and economic costs. Specifically, in 2010 it was estimated that crashes in which at least one driver was identified as being distracted resulted in the loss of $40 billion in economic costs (e.g., medical costs, legal and court costs, emergency service costs, property damage) and 123 billion in societal harm (3). Risks created by distracted drivers affect the driver, their passengers, and other road users. Basic categories of distractions, next explained, include those associated with visual, cognitive, and/or motor distractions (4). Visual distractions involve taking eyes off the road—such as looking at the radio, or staring at a crash scene. Cognitive distractions occur when the driver is not mentally focused on their driving, such as when creating a voice-to-text message. Motor distractions are tasks such as eating, drinking or grooming, while driving. Distractions may combine the three categories, such as a driver who is placing a call (motor), looking at the phone (visual), and thinking about what to say (cognitive).

In the U.S. in 2015, 10% of fatal crashes were distraction-affected, resulting in 3,477 fatalities (5). A “distraction-affected crash” indicates that one of the drivers involved was identified as distracted at the time of the crash (6). Similarly, 15% of injury crashes in 2015 were distraction affected, resulting in 391,000 injuries (5). However, the scope of the problem is likely underestimated as the assessment and on-scene crash coding to identify distraction are often lacking. Compared to other crash predictors, e.g., the influence of bad weather, determination of distraction factors is more difficult. Despite the difficulty in determining the full extent of the distracted driving problem, we do know that teen drivers represent a significant portion of distraction-impaired drivers (7).

### Teens and Distracted Driving

Teens are the group most likely to drive distracted and to suffer its consequences. Amongst drivers of all ages involved in fatal crashes, those ages 15–19 had the highest proportion of drivers judged as distracted at the time of the crash (8). For children, youth, and young adults age 8–24, motor vehicle crashes are the primary cause of death, with teens having the highest proportion of distraction-related fatalities (7).

Today's teens, to a large extent, live connected lives via computers, cell phones, and other electronic devices. Remaining engaged with others in a variety of locations and situations is for many an ingrained habit which has become the societal norm (9, 10). Reports of teen texting vary from 30 to 100 texts a day (9, 11), and over 40% of teens report texting and driving in the last 30 days, according to a Centers for Disease Control and Prevention (CDC) survey (12). In addition to using their phones, teens are more likely to engage in other types of secondary tasks which are distracting and to have longer periods of off-road glances (13). Distracted driving is risky for drivers of all ages, but research shows that these types of behaviors are riskier for novice drivers (14, 15). The proposed reasons for this are tri-fold: First, novice drivers lack skills in *hazard anticipation* and fail to fully grasp the risks involved in distracted driving (14, 15). For this reason, they may choose to engage in distracted driving in higher-risk driving situations, compared to more experienced drivers (15). Second, teens are not as experienced in *hazard mitigation*. When driving risks are experienced they do not have the skill set or the level of skill needed to avoid the hazard, or negotiate the hazard, without harm (14). Third, teens have more difficulty with *attention maintenance*. Teen drivers, often engage in other sensory activities while driving, may be challenged when they are tasked to maintain continuous engagement with a less-stimulating road environment. Thus, they may potentially respond to such a challenge by seeking stimuli that also enable distraction (15). Given the extent of the problem, and the unique factors underlying teen distracted driving as compared to adults, the need for a teen-specific distracted driving intervention is clear.

### Intervention Approaches

Documented interventions for distracted driving amongst teens have the potential to improve teen as well as societal safety (1, 14, 16–19). Such intervention approaches include those with positive (e.g., messages encouraging adoption of no phone use in car), negative (e.g., regulation of texting and driving), or adverse reinforcers (e.g., loss of driving privileges for violation of graduated driver licensing rule on passengers) for undesired behaviors and those with a mixture of the aforementioned.

In general, the effectiveness of prior interventions, i.e., translation of the interventions to preventing crashes and saving lives, with teen drivers is low (14). In response, best practices have been put forth specific to teen driver education and training, including the following:

- Using a highly appealing curriculum that is engaging and captures their attention (14);
- Training teens for new knowledge and presenting novel material and learning challenges in a tiered, development-based manner (20);
- Focusing on very specific skills such as “resistance skills” to peer influence or behind-the wheel skills in environments from parking lot to highway (14, 19);
- Providing scenarios to facilitate application of knowledge and rehearsal of driving skills (19, 21); and
- Emphasizing parent education and training in monitoring teen driving (9, 22).

Clearly, distracted driving is a public health issue and a target for intervention (23). Therefore, successful intervention plans to mitigate teen distracted driving are of critical need for teen safety and public health safety.

### Rationale and Significance

Efforts to address distracted driving must be developed and implemented in a way that targets the teen drivers' needs in the context of the regulatory environment. Such an environment includes knowledge of statewide resources and/or following procedures related to the state's highway safety, state laws, and state regulations. Currently only a few state-focused teen programs exist. One such program is IMPACT Teen Drivers available in California. The goal of this program is to address teen distracted driving and reckless driving and the content includes resources from informational handouts to videos targeting six stakeholder groups, such as teens and parents. Successes of this program are its nationwide recognition and outreach across states to include Texas and Pennsylvania, and with eight additional community partners. Because no formal program exists in the State of Florida, and in order to meet the needs and requests of the Florida Department of Transportation, and in support of the Strategic Highway Safety Plan for Florida, we synthesized the research published between 2000 and 2017, on teen distracted driving as a foundation for model program development of a computer-based training program.

### Aim

The aim of this study was to conduct an integrative review of published research and nationwide model programs on teen distracted driving. An integrative review design provides a synthesis of knowledge on a topic and address the significance of included study results for practice (24). The research question was: According to the distracted driving literature, what are the most efficacious and effective interventions to reduce the number of crashes, injuries, and/or fatalities among teen drivers in the United States?

## Methods

A preliminary review by the University of Florida Institutional Review Board indicated an integrative review did not fall under their purview.

### Design

We used an integrative review methodology because it is the most appropriate for identifying evidence from teen-distracted driving educational interventions. The five steps that guided this review included: identify the research problem, collect the literature, evaluate the literature, synthesize, and integrate the data and present the results (24, 25). Our approach provided a robust framework for data collection, analysis, and synthesis. We performed this review over two phases where the first included title and abstract screening, and the second included an in-depth full-text extraction and synthesis.

### Protocol and Registration

The integrative review framework and the *PRISMA-P 2015 checklist* guided protocol development. The health sciences librarian's (JMD) preliminary search for related integrative reviews and systematic reviews on October 30, 2017, yielded no relevant registrations in either the *JBI Database of Systematic Reviews, or Implementation Reports* or in *International Prospective Register of Systematic Reviews (PROSPERO)*. The protocol for this study was not registered, corresponding with PROSPERO's statement that review types other than systematic reviews are not eligible for inclusion (26).

### Search Methodology

The health sciences librarian conducted a systematic literature search. The base form of the search strategy was developed through the *Population, Interventions, Comparators, Outcomes*, and *Study Design* (PICOS) framework (27). Five *inclusion criteria* were: participant population included motor vehicle drivers aged 15–19; interventions were related to reducing teen driver inattention and distraction-related motor vehicle crashes, injuries, and/or fatalities; comparators were unspecified; outcomes addressed driving performance in relation to improving driver behavior, attitudes toward risk-taking, and knowledge of forms of distracted driving; and study design was experimental, quasi-experimental, systematic review, or experimental designs with prospective longitudinal cohorts, within the context of efficacious and effective intervention studies. *Additional search criteria* included publication in the English language, for the time period of January 2000 to November 2017.

Refinement of the search strategy occurred via project team recommendations, *MeSH* and keyword term testing in *PubMed* and *Web of Science*, and peer-review by a second health science librarian. During November 22–23, 2017, the final search was conducted on six bibliographic databases that had been selected as relevant to the topic of interest as displayed in Table 1. These databases included EBSCO Host's *CINAHL, PsycINFO*, ProQuest's *ERIC, PubMed* (NCBI), *Transport Research International Documentation*, and *Web of Science*. The search strategy was adapted for each database through truncation and phrase-searching (in the title and abstract fields where available), limiters (English language and 2000–2017 publication years), and subject headings (*CINAHL Headings, ERIC Descriptors, Thesaurus of Psychological Index Terms, MeSH, Transportation Research Index Terms*). Gray literature was identified within these six databases, but was limited to book chapters, conference abstracts, conference proceedings, policy documents, and white papers (28). The completed search strategy is available from the librarian.

**Table 1:** Database searched by subject, results and search date.

**Database**	**Subject**	**Results (*n*)**	**Search date**
CINAHL (EBSCOHost)	Allied health, biomedicine	17	22 Nov 2017
ERIC (ProQuest)	Education	1	23 Nov 2017
PsychINFO (EBSCO)	Behavioral science, biomedicine, health sciences	10	23 Nov 2017
PubMed (NCBI)	Biomedicine, health sciences	128	22 Nov 2017
Transport research international documentation	Transportation science	25	23 Nov 2017
Web of science core collection (Thomson Reuters)	Automation, behavioral science, computer science, engineering, health sciences, transportation sciences	4	22 Nov 2017

*Nov, November*.

### Search Outcome

A total of 185 studies, retrieved from the database searches, were exported to *Mendeley Desktop*, and de-duplicated. One-hundred and eighty-five unique studies were identified and progressed to a screening stage where titles and abstracts were retrieved and reviewed. No additional records were identified through other sources.

For agreement between raters after the full-text assessment, (*n* = 17) a perfect κ of 1.0 was reached for each dyadic group.

### Data Abstraction and Synthesis

A title and abstract screening was conducted on these 185 studies. Team members (*n* = 6) were *first* randomized into dyad pairs (i.e., *three* total groups comprising *two* researchers each). A total of 137 records, that did not match the study's inclusion criteria were excluded. Decisions of team members were analyzed in their dyad pairs for inter-rater reliability. We used Cohen's kappa coefficient (κ) to measure inter-rater agreement, which is considered a more robust measure than simple percent agreement calculation because κ considers the possibility of the agreement occurring beyond chance (29).

**Figure 1:**
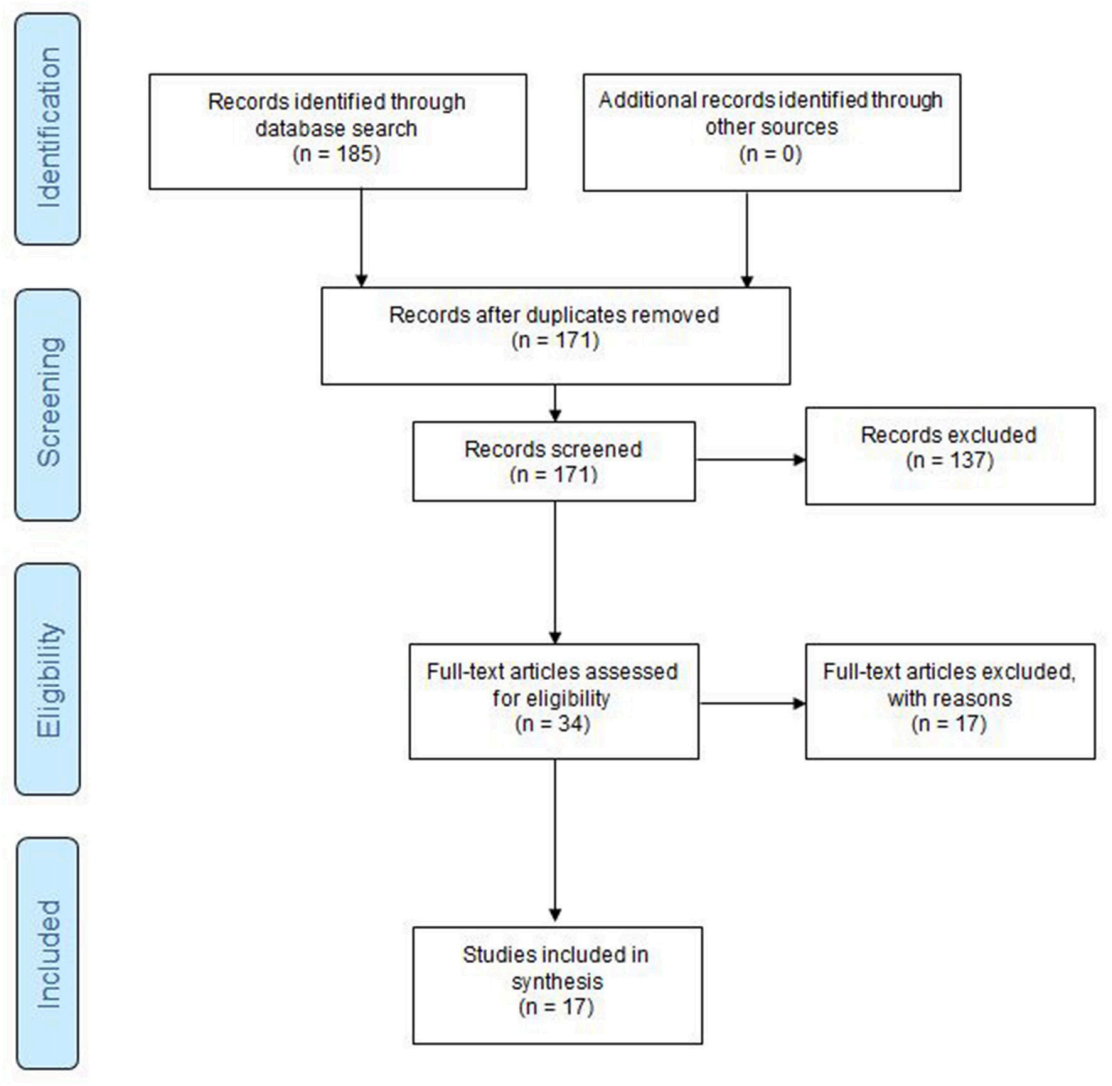
PRISMA P Flow Diagram of abstract screening and full text extraction process. Flow Diagram adopted from Moher et al. (30).

Next, a full-text evaluation was performed on the 34 remaining studies. Dyad groupings remained the same, but studies being evaluated were again randomized among the dyad pairs. We excluded 14 studies at full text evaluation stage. Subsequently, we extracted and recorded data from 17 studies using the following subheadings: reference(s), age of population, study type, purpose of study, funding source, location of study, gender characteristics, sample size(s), intervention type(s), study outcomes, study comparisons, intervention duration, effect size(s), effectiveness of interventions, and study limitations. Figure 1 illustrates the process of abstract and full text screening.

### Critical Appraisal

Copper (31) suggests that extraction of specific methodological characteristics of primary studies could be used to evaluate overall quality of the studies. The Joanna Briggs Institute provides a *Critical Appraisal Checklist for Randomized Controlled Trials* (32). As such, we used a combination of the aforementioned approaches to appraise the studies.

## Results

### Inter-rater Reliability

The first round of reviews indicated fair agreement with Cohen's kappa (κ) = 0.31–0.34 (0.21–0.40 is considered fair) (29). For this review, the a-priori determined kappa was >0.81. Therefore, re-screening occurred among the groups to arrive at complete agreement with κ = 1.0.

### Descriptive Profile of the Primary Studies

The 17 experimental studies that underwent critical review, is displayed in Table 2.

**Table 2:** Summary of intervention types separated by experimental designs.

**Intervention type (*N* = 17)**	**Experimental design**	**Article**
1. Presentations, videos, brief instructional programs (*n* = 5)	RCT	(33, 34)
	Pre-post	(35, 36)
	Longitudinal	(37)
2. Education or training programs (*n* = 2)	Prospective cohort	(38)
	Quasi-experimental	(39)
3. Driving simulator (*n* = 2)	RCT	(40, 41)
4. In-vehicle monitoring or feedback studies (*n* = 3)	RCT	(42, 43)
	Pre-post	(44)
5. Integrated programs (*n* = 5)	RCT	(45, 46)
	Pre-post	(47)
	RCT	(48, 49)

*RCT, Randomized control trial*.

These studies all comprised subjects 15- to 19-years old, had experimental designs which included randomized controlled trials (RCT) (*n* = 10), pre-post designs (*n* = 4), quasi-experimental trial (*n* = 1), longitudinal follow up from an experimental design (*n* = 1) and a prospective cohort following an experimental design (*n* = 1). While these studies generally examined distracted driving interventions and effectiveness, heterogeneity was observed in the intervention type, as next described.

### Interventions

The studies were thematically characterized in relation to the intervention type and delivery methods to help inform our model program in development. Table 2 indicates the five categories of interventions employed by design: presentations, videos, or brief instructional programs (*n* = 5) (33–37); education or training programs (*n* = 2) (38, 39); driving simulators (*n* = 2) (40, 41); in-vehicle monitoring or feedback studies (*n* = 3), (42–44); and integrated programs (*n* = 5) (45–49).

### Delivery Methods

Since delivery methods were combined in studies, several studies adopted more than one delivery method. However, the majority of interventions utilized educational trainings or presentations (34, 35, 37–39, 45, 48, 49). These methods are easier to present in multiple locations and to larger groups. Alternatively, on-road assessments via self-evaluation or driving performance feedback (33, 42–44, 46, 47) and driving simulation interventions (40, 45–49) provided the ability to learn skills in a real-world setting or simulated environment representative of the real world. Interventions that were PC-based, web-based, or through an e-learning platform had the benefit of remote delivery to teens, parents and other users. Moreover, these modes of delivery accommodated the time, location, and pace of instruction to the participants (33, 46, 47). 36 examined use of instructional videos. Benefits of this mode include that it provides consistent content delivery in a cost-effective way that is not reliant on in-person instruction. Three studies using persuasive messaging leveraged this strategy to shift the attitudes of teens against distracted driving behaviors (33, 35, 39).

At healthcare facilities education teams could work with health care professionals who had a first-hand knowledge of the injury outcomes of distracted driving. Healthcare facilities are uniquely positioned to be able to offer trauma survivor testimony or a mock trauma session (35, 38); and to reinforce experiences with a hospital or trauma center tour (35). Two studies focused on including peers or parents in a peer-generated anti-texting program (39), or a parent-teen driving agreement (34). Messages delivered by peers and parents are recognized as more effective compared to interventions who did not include peers or parents.

### Integrated Review Findings

Table 3 displays results from the 17 included studies by design type, authors (year), study details, sample size, effectiveness and funding. The sample sizes ranged from small (*N* = 12) to large (*N* = 1,365). Although not always clearly documented in the outcome of the primary studies, the research team categorized the outcomes of each included study in terms of efficacy or effectiveness, with 100% agreement among team members. That is the team assessed if their study outcomes aligned with our outcomes of interest (i.e., teen crashes, injuries, or fatalities as well as driver ability, skill or performance). Of the 17 studies, and based on their findings as published, we categorized studies as effective (or not) or efficacious (or not), or indicate when this detail was not stated. Only 15 of the 17 studies reported funding from state and federal highway traffic safety entities, National Safety Council, associations for law enforcement, teacher associations, or healthcare corporations, among others.

**Table 3:** Study summaries by design type, author, study details, sample size, effectiveness, funding and outcomes.

**Design type and authors (Year)**	**Study details**	**Sample size (totals)**	**Effectiveness**	**Funding**
**RANDOMIZED CONTROLLED TRIAL**				
Campbell et al. (40)	Purpose: To determine if driving simulator training lowers motor vehicle crash rates for novice teen drivers Intervention type: Driving simulation Delivery method(s): Tutorial, simulator, survey	215	Effective Outcome: No No significant differences were seen between the intervention and control groups for simulator training in MVC (*p* > 0.05) and driving infractions (*p* < 0.05). Effect size or SE: Not applicable	Yes
Cox et al. (41)	Purpose: To quantify the effect of stimulants on driving performance of young adult drivers Intervention type: Driving simulation Delivery method(s): Simulator	35	Effective Outcome: Yes Overall, stimulant use was associated with better driving performance (*F* = 7.16, *p* < 0.001). Effect size or SE: Not indicated	Yes
Jemakian et al. (42)	Purpose: To determine if the presence of collision warning systems alter teenagers' driving as measured by headway maintenance, lane change/lateral drift, and signal use? Intervention type: In-vehicle monitoring or feedback study Delivery method(s): In-vehicle warning system	40	Effective Outcome: Yes Collisions warning system use was associated with increased signal use and a 37% reduction in lane drift warnings (*p* < 0.001). Effect size or SE: Not indicated	Yes
Knodler and Fisher (48)	Purpose: To determine whether the SAFE T program will prove effective among novice drivers Intervention type: Integrated program Delivery method(s): Training program, simulator evaluation	48	Effective Outcome: Yes Results report suggest the SAFE T program may improve hazard anticipation [*t*_(30)_ = 2.41, *p* = 0.022]., hazard mitigation [*t*_(29)_ = 2.34, *p* = 0.028] and attention maintenance [*t*_(30)_ = 2.74, *p = 0*.010] in teen drivers. Effect size or SE: Not indicated	Yes
Krishnan et al. (45)	Purpose: To determine if hazard anticipation training helped young drivers improve their strategic engagement in secondary tasks in the presence of latent hazards Intervention type: Integrated program Delivery method(s): Training program, simulator evaluation	12	Effective Outcome: Yes Overall, hazard anticipation training indicated an increase in the portion of latent hazards detected with significant main effects for type of treatment and environment (Wald X${}_{1}^{2}$ = 264.66, *p* < 0.01). Effect size or SE: Not indicated	Yes
Mirman et al. (33)	Purpose: To identify the mechanisms by which the TeenDrivingPlan may be effective and to extend our understanding of how teens learn to drive Intervention type: Presentation, video, survey, or brief instructional program Delivery method(s): Web-based intervention, videos	151 teen/parent dyads	Effective Outcome: Yes Results indicated exposure to the TeenDrivingPlan increased teen's perceived social support (*M* = 4.11, *SD* = 0.73, *p* = 0.01), parent engagement (*M* = 3.51, *SD* = .54, *p* = 0.03) and practice diversity (*M* = 6, *IQR* = 5–6, *p* = 0.01). Effect size or SD: Not indicated	Yes
Simons-Morton et al. (43)	Purpose: To determine the extent to which two forms of feedback (Lights only, Lights plus delayed feedback) altered elevated g-force event rates among novice teen drivers Intervention type: In-vehicle monitoring or feedback study Delivery method(s): On-road feedback, survey	88 teen/parent dyads	Effective Outcome: Yes Overall, teens in the Lights Plus group had fewer driving events (slope = −0.11, *p* < 0.01) than the lights only group (slope = 0.05, *p* = 0.67). Effect size: 1.67, favoring the Lights Plus group	Yes
Thomas et al. (46)	Purpose: To describes the methods and results of three coordinated studies related to the development of a PC-based attention maintenance training program and its evaluation on a computer, in the field, and in a high-fidelity driving simulator. Intervention type: Integrated program Delivery method(s): Eye tracking system, on-road evaluation, driving simulator	Study 1 = 30 Study 2 = 37 Study 3 = 40	Effective Outcome: Yes for studies 1 and 2 but not 3 Study 1: For the intervention group, the duration of glances less than 7 s decreased significantly [*t*_(38)_ = 1.912, *p* = 0.077] Effect size or SE: SE was 0.025 Study 2: Teens in intervention groups had significantly lower proportions of off-road glances while performing non-driving tasks than the placebo group for 2 s [*t*_(38)_ = 2.28, *p* = 0.029] and 2.5 s [*t*_(38)_ = 2.27, *p* = 0.030] glances. Effect size or SE: Not indicated Study 3: Overall, the time spent glancing off the road for more than 2.0 s [*t*_(38)_ = 2.99, *p* = 0.005]., 2.5 s [*t*_(38)_ = 4.2, *p* < 0.001], 3.0 s [*t*_(38)_ = 2.75, *p* = 0.009] and maximum glances off the road [*t*_(38)_ = 2.42, *p* = 0.021] decreased significantly. Effect size or SE: Not indicated	Yes
Yamani et al. (49)	Purpose: To determine the effectiveness of a novel integrated training program (SAFE-T) and determine if integrating the training of all the three higher cognitive skills would yield results comparable to existing programs Intervention type: Integrated program Delivery method(s): Training program, simulator evaluation	48	Effective Outcome: Yes Overall, results indicated there was an effect of for hazard anticipation training (*F* (2,45) = 4.00, *p* = 0.025*, MSE* = 3.90). No statistical differences existed between the groups for hazard training (*p* > 0.005). There was an overall training effect for attention maintenance [*F*_(2, 45)_ = 4.00, *p* = 0.042, *MSE* = 0.876]. Effect size or SE: Not indicated	Yes
Zakrajsek et al. (34)	Purpose: To test the effectiveness of the Checkpoints program when delivered to parents/adolescents by driver education instructors Intervention type: Presentation, video, survey, or brief instructional program Delivery method(s): Group discussion, driving agreement	148 teen/parent dyads	Effective Outcome: Yes Teens in the intervention group reported less; overall risky driving (intervention = 0.50; control = 0.82, *p* = 0.04), frequency of driving 20 mph over the speed limit (intervention = 0.02, control = 0.28, *p* = 0.02), and driving through yellow lights (intervention = 1.79, control = 3.15, *p* = 0.04). Effect size or SE: Not indicated	Yes
**PRE-POST**				
Adeola et al. (35)	Purpose: To examine the effect of the “Get the Message: A Teenage Distracted Driving Program” on changes in driving behaviors, attitude, and knowledge. Intervention type: Presentation, video, survey, or brief instructional program Delivery method(s): Video, survey	1,238	Effective Outcome: Yes After completion of the program, participants who believed texting and driving was a crash risk increased from 29 to 77% (*p* < 0.001). Effect size or SE: Not indicated	No
Kidd and Buonarosa (44)	Purpose: To determine if warnings from an integrated safety system provided a negative reinforcement contingency that decreases the overall likelihood that drivers engage in various secondary behaviors or increases likelihood that drivers engage in secondary behaviors due to perceived safety benefits Intervention type: In-vehicle monitoring or feedback study Delivery method(s): In-vehicle warning	40 teens and 108 adults	Effective Outcome: No No significant differences existed between pre and post-test for the secondary behaviors examined, age and vehicle speed (*p* > 0.05). Effect size or SE: Not applicable	No
King et al. (36)	Purpose: To determine if the “You Hold the Key” teen driving countermeasure increase seat belt use and decrease drunk driving or riding with a drunk driver? Intervention type: Presentation, video, survey, or brief instructional program Delivery method(s): Survey	1,365	Effective Outcome: Yes Overall, compared to pre-test data, participants immediately following the program and 6 months later reported to be significantly more likely to report wearing their seatbelts (*MD* = 0.18, *p 0*.001); requiring their passengers to wear seatbelts (*MD* = 0.48, *p* < 0.001); limiting the number of passengers to number of seatbelts (*MD* = 0.54 *p* < 0.001); avoiding drinking/driving situations (*MD* = 0.21, *p* < 0.001); and saying no to riding with a friend who had been drinking and planning to drive (*MD* = 0.16, *p* < 0.001). Effect size or SE: not indicated	No
National Highway Traffic and Safety Administration (47)	Purpose: To describe the mehods and results of three studies that developed and evaluated the Forward Concentration and Attention Learning (FOCAL) training program Intervention type: Integrated program Delivery method(s): PC-based evaluation, on-road evaluation, simulator	Study 1 = not indicate Study 2 = ~40Study 3 = not indicated	Effective Outcome: Yes Study 1: Results reported FOCAL training reduced the duration of off road glances < 7 s [*t*_(38)_ = 1.912, *p* = 0.077] Effect size or SE: SE was 0.025 Study 2: The FOCAL group had lower proportions of off-road glances while performing non-driving tasks than the placebo group for 2 s [*t*_(38)_ = 2.28, *p* = 0.029] and 2.5 s [*t*_(38)_ = 2.27, *p* = 0.030] glances. Effect size or SE: Not indicated The percentage of drivers that looked away once for at least more than 2.0 s [*t*_(38)_ = 2.99, *p* = 0.005]., 2.5 s [*t*_(38)_ = 4.2, *p* < 0.001], and 3.0 s [*t*_(38)_ = 2.75, *p* = 0.009] and maximum glances off the road [*t*_(38)_ = 2.42, *p* = 0.021] decreased significantly. Effect size or SE: Not indicated	Yes
**QUASI-EXPERIMENTAL**				
Unni et al. (39)	Purpose: To assess the effectiveness of a hospital school program on students' knowledge and behaviors regarding texting while driving Intervention type: Education or training program Delivery method(s): Hospital/School education, peer designed program	Phase 1 = 137 Phase 2 = not indicated	Effective Outcome: Yes Students knowledge of driving licensing laws and feelings of empowerment to take action with a teen driver who was texting significantly increased (χ^2^ = 65.787, *df* = 1, *p* < 0.001). Overall, the proportion of adults and teen drivers texting at baseline significantly decreased from 13 to 10% and 12 to 9%, respectively, marking a significant decrease (*p* < 0.0001). Effect size or SE: not indicated	Yes
**LONGITUDINAL**				
Manno et al. (37)	Purpose: To determine the driving offense recidivism rates for Teen RIDE participants vs. the control group Intervention type: Presentation, video, survey, or brief instructional program Delivery method(s): Discussion groups	268	Effective Outcome: Yes The driving offense recidivism rate for teens in the RIDE program (5.8%) compared to the control group (55.8%) significantly decreased (*p* < 0.001). Effect size or SE: not indicated	No
**PROSPECTIVE COHORT**				
Ekeh et al. (38)	Purpose: To compare the traffic offense recidivism rate of adolescents who had completed the Drive Alive Program and those who had not Intervention type: Education or training program Delivery method(s): Interactive education; trauma survivor; mock trauma session	946	Effective Outcome: Yes The traffic offense recidivism rate for adolescents who completed the program (26.4%) 6 months post program compared to the control group (32.3%) significantly decreased (*p* = 0.038). Effect size or SE: Not indicated	Not indicated

*Df, degrees of freedom; F, F-test; IQR, interquartile range; M, mean; MD, mean difference; MSE, mean squared error; MVC, motor vehicle collisions; SD, standard deviation; SE, standard error; RCT, randomized control trial; χ^2^, chi Square*.

### Interventions Studied by Type of Experimental Design

Ten of the 17 studies were RCTs, of which all but 1 presented results supporting intervention effectiveness. The most common effective interventions, documented in five studies, focused on one or more of three critical skills underpinning driving: i.e., hazard awareness, hazard mitigation, and attention maintenance (45–49).

Interventions deploying in-vehicle technology, for monitoring or feedback, were less common but two RCTs provided evidence in favor thereof (42, 43). Interventions effective for on-road outcomes of teens' driving behavior targeted a RCT with training content to establish parent/teen driving contract (34), or web-based instructional modules for parents/teens that focused on knowledge for novice drivers and provided teen driving practice guidance (33). A quasi-experimental design was used to study a hospital/school partnership for an educational campaign (39). The campaign targeted teens and adults, who were primarily teachers. This campaign was effective on outcomes of empowering teens to stop others' texting, as well as to curtail personal texting. School observation of drivers' texting behavior at the end of campaign demonstrated an overall decrease of drivers' texting from 13 to 10% with a greater difference for teens vs. adults. Studies that provided support for effectiveness of educational programs among juvenile traffic offenders included a longitudinal study (37) and a prospective cohort study (38). One unique study addressed a sub-population of teens, those with Attention Deficit Hyperactivity Disorder (ADHD), and an intervention of stimulant medication. For teens with ADHD, Cox et al. (41) RCT found that stimulant use, compared to placebo, was associated with improved driving (e.g., more time spent on the road vs. shoulder, smoother braking, less speeding, better speed consistency, appropriate use of brakes, and slower more controlled turns).

Only two interventions were not shown to be effective. The studies with negative results included an RCT study of simulator training with outcomes of motor vehicle crash occurrence or driving infractions in the 6–12 months post-intervention (40), as well as a pre-post study examining the effectiveness of warnings from in-vehicle monitoring measuring engagement in secondary behaviors (i.e., driving distractions) as outcomes (44).

An integration of the results above suggest that a model program to decrease teen distracted driving may contain the following elements: First, hazard awareness, hazard mitigation, and attention maintenance are essential ingredients for curbing distracted driving. Second, in-vehicle technology, for monitoring or feedback, contributes to the success of driving without distraction. Third, a parent/teen driving contract is essential for curtailing distracted driving behaviors. Fourth, partnerships among organizations have beneficial outcomes to mitigate forms of distracted driving, if the partners provide support of a targeted educational campaign addressing such behaviors. Finally, effectiveness for educational programs exist for juvenile traffic offenders (37, 38), or when teens with medical conditions drive with optimal therapeutic dosages of their medications (41).

## Discussion

By reviewing and extracting data from 17 studies, we synopsized interventions for distracted driving and recorded outcomes of efficacy or effectiveness. Interventions commonly targeted three elements of driving: hazard awareness, hazard mitigation, and attention maintenance (15). Improvements in these skills were confirmed by interventions targeting one [i.e., hazard anticipation, (45)] or all three of these skills [e.g., Yamani et al. (49)].

The theme of using technology was present in about half of the studies. For example, simulators were used in 7 of the 17 studies as either as a training tool, or as a method of measuring driving performance. Moreover, in-vehicle warnings were also successfully used as interventions to shape teen's driving behaviors such as lane maintenance (42) or smoother acceleration/braking (43).

We identified parent/adult driver involvement as an important attribute to the success of the intervention. Specifically, parent/adult driver involvement lends itself to an educational intervention strategy to improve carry-over of learning, as well as improving the quality and diversity of driving practice (12, 19).

Finally, partnerships among organizations may have beneficial outcomes to mitigate forms of distracted driving, if the partners provide support to a targeted educational campaign addressing such behaviors (39). This finding has specific application for our model educational program in development. That is a computer based training (CBT) program, with collaboration between the Florida Department of Transportation and the University of Florida will target mitigating distracted driving in teens. Potential opportunities also exist to target juvenile offenders, with an expectation of successfully curbing distracted driving after exposure to the CBT.

The last 40 years have seen a downturn in driver education as a component of secondary education in the U.S. and as a priority of NHTSA (20). As new driver education programs and strategies are being developed, research, as is the case in this integrated review, must guide state and national initiatives in the use of the most efficacious and effective strategies. Such strategies may be beneficial in curbing fatalities and injuries from teen distracted-driving crashes.

### Limitations

While several of the studies reported effectiveness, only one, the FOCAL program (47) used a longitudinal design and showed long-term outcomes in favor of reduced teen crash risk. Several study authors noted unequal sample distribution in including studies with only males, studies not representing the age range of novice drivers, and studies lacking diversity of race or ethnic background of participants. These types of sampling limitations can compromise internal validity as well as generalizability of results. Generalizability of results may also be a limitation of driving simulator studies or of studies conducted in a particular geographic area. As shown by the breakdown of studies, lack of randomization was a limitation for some studies, and other studies reported low levels of participation and/or attrition that occurred if the study protocol had multiple sessions. The studies were subject to multiple forms of bias. This includes selection bias, i.e., convenience sampling; self-report bias, i.e., asking teens to report their driving behaviors; spectrum bias, i.e., failing to test relevant sub-groups in a study; Hawthorne bias, i.e., behavior may change when observed; learning effects bias, i.e., use of the same simulator scenarios; and order effects, i.e., using the same simulator scenarios without randomization of scenes. We recognize that distracted driving is multi-faceted, however our focus was on observed behaviors representing distraction. Thus, medical conditions, drug use, and/or alcohol use impacting distraction were not examined independently. We included teens between the ages of 15–19 years as a “homogenous group.” Although we acknowledge that heterogeneity and variability exists for driving performance, skill and experience.

### Strengths

This review was conducted by an experienced team who followed the PICOS framework and guidance from the Joanna Briggs Institute on conduct of a review (27). The search strategy was implemented by a health science center librarian and studies were selected using clear inclusion and exclusion criteria. A rigorous measure of reliability was used to assess the level of consistency among dyad pairs to include/exclude studies from the abstract and full text reviews. An iterative team approach ensured consensus among team members during all aspects of the integrated review process.

### Implications/Future Directions

This review, and the related findings, contributes to developing an evidence-based CBT intervention on teen distracted driving. As such, these findings highlighted the state of empirical knowledge on distracted driving interventions to our stakeholders (i.e., Florida Department of Transportation State Safety Office, advocacy groups for teen driving safety, and teen drivers themselves).

The integrative review identified the vital link between skills training and crash reduction as well as the effectiveness of computer-based interventions. Further, the review suggested an appropriate outline and approach. This includes providing teens with an informational foundation followed by skills training in the areas of hazard awareness, hazard mitigation, and attention maintenance. This occurs within a framework that balances a positive informational approach with an appropriately cautionary approach. Moreover, a CBT will be supported by two organizations, the Florida Department of Transportation and the University of Florida, with a mission driven focus to decrease and prevent teen distracted driving.

## Author Contributions

SC developed and designed the review. JM-D carried out the database search for the integrative review. CB, NA, SC, SW, and JM-D assisted with conducting the review and integrating findings. SC wrote the manuscript with support from SW, JM-D, and SM.

### Conflict of Interest Statement

The authors declare that the research was conducted in the absence of any commercial or financial relationships that could be construed as a potential conflict of interest.

## References

[1] National Highway Traffic and Safety Administration Driver Distraction: A Review of the Current State-of-the-Knowledge. (DOT HS 810 787). Washington, DC: U.S. Department of Transportation (2008).

[2] National Highway Traffic Safety Administration Traffic safety facts 2012: Young drivers. Washington, DC: Department of Transportation (2012).

[3] Blincoe LJ Miller TR Zaloshnja E Lawrence BA The Economic and Societal Impact of Motor Vehicle Crashes 2010. (Revised) (Report No. DOT HS 812 013). Washington, DC: National Highway Traffic Safety Administration (2015).

[4] Vegega M Jones B Monk C Understanding the Effects of Distracted Driving and Developing Strategies to Reduce Resulting Deaths and Injuries: A Report to Congress. Washington, DC: National Highway Traffic Safety Administration (2013).

[5] National Highway Traffic and Safety Administration Traffic Safety Facts - Research Note, Distracted Driving 2015. (DOT HS 812 381). Washington, DC National Highway Traffic and Safety Administration (2017).

[6] National Traffic Law Center Investigation and Prosecution of Distracted Driving Cases (DOT HS 812 407). Washington, DC: National Highway Traffic Safety Administration (2017).

[7] National Highway Traffic and Safety Administration Traffic Safety Facts - Research Note, Motor Vehicle Crashes as a Leading Cause of Death in the United States, 2015. (DOT HS 812 499). Washington, DC: National Highway Traffic and Safety Administration (2018).

[8] National Highway Traffic and Safety Administration Trafic Safety Facts: Distracted Driving 2011 (DOT HS 811 737). Washington, DC: National Highway Traffic and Safety Administration (2013).

[9] Delgado MK Wanner KJ McDonald C. Adolescent cellphone use while driving: an overview of the literature and promising future directions for prevention. Media Commun. (2016) 4:79–89. 10.17645/mac.v4i3.536 27695663 PMC5041591

[10] Foss RD Goodwin AH. Distracted driver behaviors and distracting conditions among adolescent drivers: findings from a naturalistic driving study. J Adolesc Health. (2014) 54:S50–60. 10.1016/j.jadohealth.2014.01.005 24759441

[11] Lenhart A Teens Social Media & Technology Overview 2015: SMARTPHONES Facilitate Shifts in Communication Landscape for Teens. Washington DC (2015). Available online at: www.pewinternet.org/2015/04/09/teens-social-media-technology-2015

[12] OlsenEOShultsRAEatonDK. Texting while driving and other risky motor vehicle behaviors among US high school students. Pediatrics. (2013) 131:e1708–15. 10.1542/peds.2012-346223669511

[13] Simons-Morton BG Guo F Klauer SG Ehsani JP Pradhan AK. Keep your eyes on the road: young driver crash risk increases according to duration of distraction. J Adolesc Health. (2014) 54:S61–7. 10.1016/j.jadohealth.2013.11.021 24759443 PMC3999409

[14] Buckley L Chapman RL Sheehan M. Young driver distraction: State of the evidence and directions for behavior change programs. J Adolesc Health. (2014) 54(Suppl. 5):S16–21. 10.1016/j.jadohealth.2013.12.021 24759436

[15] Carter PM Bingham CR Zakrajsek JS Shope JT Sayer TB. Social norms and risk perception: predictors of distracted driving behavior among novice adolescent drivers. J Adolesc Health. (2014) 54(Suppl. 5):S32–41. 10.1016/j.jadohealth.2014.01.008 24759439 PMC7189891

[16] Carey RN Sarma KM. Threat appeals in health communication: messages that elicit fear and enhance perceived efficacy positively impact on young male drivers. BMC Public Health. (2016) 16:1–16. 10.1186/s12889-016-3227-2 27460475 PMC4962518

[17] Layba C Griffin LW Jupiter D Mathers C Mileski W. Adolescent motor vehicle crash prevention through a trauma center-based intervention program. J Trauma Acute Care Surg. (2017) 83:850–3. 10.1097/TA.0000000000001605 28557846

[18] Merrikhpour M Donmez B. Designing feedback to mitigate teen distracted driving: a social norms approach. Acci Anal Prevent. (2017) 104:185–94. 10.1016/j.aap.2017.04.016 28544953

[19] Winston FK Mirman JH Curry AE Pfeiffer MR Elliott MR Durbin DR. Engagement with the TeenDrivingPlan and diversity of teens' supervised practice driving: Lessons for internet-based learner driver interventions. Injur Prevent. (2015) 21:4–9. 10.1136/injuryprev-2014-041212 24916684

[20] Mayhew DR. Driver education and graduated licensing in North America: past, present, and future. J Safety Res. (2007) 38:229–35. 10.1016/j.jsr.2007.03.001 17478193

[21] Pollatsek A Narayanaan V Pradhan A Fisher DL. Using eye movements to evaluate a PC-based risk awareness and perception training program on a driving simulator. Hum Factors. (2006) 48:447–64. 10.1518/001872006778606787 17063961

[22] Dellinger AM West BA. Health care providers and teen driving safety: topics discussed and educational resources used in practice. Am J Lifestyle Med. (2014) 9:451–6. 10.1177/1559827614554903 26740816 PMC4699318

[23] Lee JD. Dynamics of driver distraction: the process of engaging and disengaging. Ann Adv Automot Med. (2014) 58:24–32. 24776224 PMC4001670

[24] de Souza MT da Silva MD de Carvalho R Integrative review: what is it? How to do it? Einstein. (2010) 8:102–6. 10.1590/s1679-45082010rw1134 26761761

[25] Hopia H Latvala E Liimatainen L. Reviewing the methodology of an integrative review. Scand J Caring Sci. (2016) 30:662–9. 10.1111/scs.12327 27074869

[26] Universty of York Centre for Reviews and Dissemination About PROSPERO: Inclusion Criteria. (2008). Available online at: www.crd.york.ac.uk/prospero/#aboutpage

[27] University of York Centre for Reviews and Dissemination Systematic Reviews: CRD's Guidance for Undertaking Reviews in Health Care (York, UK) (2009).

[28] Cooper HM Hedges LV The Handbook of Research Synthesis. New York, NY: Russell Sage Foundation (1994).

[29] Cohen J A coefficient of agreement for nominal scales. Educ Psychol Measure. (1960) 20:37–46. 10.1177/001316446002000104

[30] Moher D Liberati A Tetzlaff J Altman DG the PRISMA Group. Preferred reporting items for systematic reviews and meta-analyses: the PRISMA statement. Annals of Internal Med. (2009). 151:264–9. 10.7326/0003-4819-151-4-200908180-00135 19622511

[31] Cooper HM Integrating Research: A Guide for Literature Review, 2nd ed. Newbury Park, CA: Sage Publications (1989).

[32] Aromataris E Fernandez R Godfrey C Holly C Kahlil H Tungpunkom P. Summarizing systematic reviews: methodological development, conduct and reporting of an umbrella review approach. Int J Evid Based Healthcare. (2015) 13:132–40. 10.1097/XEB.0000000000000055 26360830

[33] Mirman JH Albert WD Curry AE Winston FK Fisher Thiel MC Durbin DR. TeenDrivingPlan effectiveness: the effect of quantity and diversity of supervised practice on teens' driving performance. J Adolesc Health. (2014) 55:620–6. 10.1016/j.jadohealth.2014.04.010 24925492

[34] Zakrajsek JS Shope JT Greenspan AI Wang J Bingham CR Simons-Morton BG. Effectiveness of a brief parent-directed teen driver safety intervention (checkpoints) delivered by driver education instructors. J Adolesc Health. (2013) 53:27–33. 10.1016/j.jadohealth.2012.12.010 23481298 PMC4147835

[35] Adeola R Omorogbe A Johnson A. Get the message: a teen distracted driving program. J Trauma Nursing. (2016) 23:312–20. 10.1097/JTN.0000000000000240 27828882

[36] King KA Vidourek RA Love J Wegley S Alles-White M. Teaching adolescents safe driving and passenger behaviors: effectiveness of the you hold the key teen driving countermeasure. J Safety Res. (2008) 39:19–24. 10.1016/j.jsr.2007.10.006 18325412

[37] Manno M Maranda L Rook A Hirschfeld R Hirsh M. The reality of teenage driving: the results of a driving educational experience for teens in the juvenile. J Trauma Acute Care Surg. (2012) 73:S267–72. 10.1097/TA.0b013e31826b00f4 23026966

[38] Ekeh AP Hamilton SB D'Souza C Everrett E McCarthy MC. Long-term evaluation of a trauma center-based juvenile driving intervention program. J Trauma. (2011) 71:223–6. 10.1097/TA.0b013e31821cc0fd 21818028

[39] Unni P Estrada CM Chung DH Riley EB Worsley-Hynd L Stinson N A multiyear assessment of a hospital-school program to promote teen motor vehicle safety. J Trauma Acute Care Surg. (2017) 83:289–95. 10.1097/TA.0000000000001521 28422920

[40] Campbell B Borrup K Derbyshire M Rogers SA Lapidus G. Efficacy of driving simulator training for novice teen drivers. Connect Med. (2016) 80:291–6. 27328578

[41] Cox DJ Merkel L Moore M Thorndke F Muller C Kovatchev B. Relative benefits of stimulant therapy with OROS methylphenidate versus mixed amphetamine salts extended release in improving the driving performance of adolescent drivers with attention-deficit/hyperactivity disorder. Pediatrics. (2006) 118:704–10. 10.1542/peds.2005-2947 16950962

[42] Jemakian JS Bao S Buonarosa ML Sayer JR Farmer CM Effects of an integrated collision warning system on teenage driver behavior. J Safety Res. (2017) 61:65–75. 10.1016/j.jsr.2017.02.013 28454872

[43] Simons-Morton BG Bingham CR Ouimet MC Pradhan AK Chen R Barretto A. The effect on teenage risky driving of feedback from a safety monitoring system: a randomized controlled trial. J Adolesc Health. (2013) 53:21–6. 10.1016/j.jadohealth.2012.11.008 23375825 PMC3644526

[44] Kidd DG Buonarosa ML. Distracting behaviors among teenagers and young, middle-aged, and older adult drivers when driving without and with warnings from an integrated vehicle safety system. J Safety Res. (2017) 61:177–85. 10.1016/j.jsr.2017.02.017 28454863

[45] Krishnan A Samuel S Dündar C Romoser M Fisher D Evaluation of a Hazard Anticipation Training Program (STRAP) on secondary task engagement in high risk scenarios. In: Paper Presented at the Transportation Research Board 94th Annual Meeting. Washington, DC (2015).

[46] Thomas FD Pollatsek S Pradhan A Divekar G Blomberg RD Reagan I Field and Simulator Evaluations of a PC-Based Attention Maintenance Training program (DTNH22-05-D-35043). Washington, DC: National Highway Traffic and Safety Administration (2011).

[47] National Highway Traffic and Safety Administration NHTSA Computer Training Program Improves Teen Drivers' Attention to the Road (408). Washington, DC: National Highway Traffic and Safety Administration (2011).

[48] Knodler MA Fisher D Evaluating the Effects of Integrated Training on Minimizing Driver Distraction (UMAR24-22). Cambridge, MA: New England University Transportation Center (2015).

[49] Yamani Y Samuel S Knodler MA Fisher DL. Evaluation of the effectiveness of a multi-skill program for training younger drivers on higher cognitive skills. Appl Ergonom. (2016) 52:135–41. 10.1016/j.apergo.2015.07.005 26360204

